# Physical activity and executive function in Chinese preschool children: the mediating role of sleep problems

**DOI:** 10.3389/fpsyg.2025.1606776

**Published:** 2025-08-19

**Authors:** Xiaolong Chen, TianChang Li, Weiling Zhou, Lancheng Huang, Sanhua Zhang

**Affiliations:** ^1^Zhejiang Philosophy and Social Science Laboratory for Research in Early Development and Childcare, Hangzhou Normal University, Hangzhou, China; ^2^Jing Hengyi School of Education, Hangzhou Normal University, Hangzhou, China; ^3^School of Psychology, Capital Normal University, Beijing, China; ^4^Wenhan Kindergarten Education Group of Hangzhou Qiantang District, Hangzhou, China; ^5^Yuncheng Kindergarten of Hangzhou West Lake District, Hangzhou, China

**Keywords:** physical activity, executive function, preschool children, sleep problems, Chinese

## Abstract

**Objective:**

This study aimed to examine the relationship between physical activity and executive function in preschool children, with a particular focus on the mediating role of sleep problem.

**Methods:**

A total of 159 preschool children participated in this study. Physical activity levels were objectively measured using the ActiGraph GT3X-BT. Sleep problems and executive function were assessed using the Chinese versions of the Children’s Sleep Habits Questionnaire and the Behavior Rating Inventory of Executive Function-Preschool Version. Statistical analyses including correlation and mediation analyses were conducted using SPSS 29.0 and Mplus 8.0.

**Results:**

(1) Light physical activity (LPA) (*r* = −0.53, *p* < 0.01), moderate-to-vigorous physical activity (MVPA) (*r* = −0.61, *p* < 0.01), and total physical activity (TPA) (*r* = −0.64, *p* < 0.01) showed significantly negatively correlated with executive function scores. (2) LPA (*r* = −0.27, *p* < 0.01), MVPA (*r* = −0.29, *p* < 0.01), and TPA (*r* = −0.31, *p* < 0.01) were significantly negatively correlated with sleep problems. (3) Sleep problems were significantly positively correlated with executive function scores (*r* = 0.47, *p* < 0.01). (4) Sleep problems mediated 24.65–28.20% of the effects of LPA, MVPA, and TPA on executive function.

**Conclusion:**

Higher levels of LPA, MVPA, and TPA were significantly associated with better executive function and fewer sleep problems in preschool children. Sleep problems play a partial mediating role in the relationship between physical activity and executive function. These findings suggest that preschool educators and parents should implement appropriate physical activity interventions to improve sleep health, ultimately fostering optimal executive function development in young children.

## 1 Introduction

Executive function (EF) refers to an individual’s ability to coordinate and regulate cognitive processes, behaviors, and emotions to achieve specific goals (Diamond and Ling, 2016). As a pivotal indicator of preschool cognitive development, EF comprises three core components: working memory, inhibitory control, and cognitive flexibility (Perone et al., 2021). With advancements in neurocognitive science, EF development and its impact on daily life have received increasing attention. The preschool years (ages 3–6) represent a critical period for prefrontal cortex maturation and EF development (Best and Miller, 2010), shaping learning abilities, social interactions, and overall well-being. Moreover, EF during early childhood exerts profound long-term effects on academic achievement and quality of life (Shokrkon and Nicoladis, 2022; Wolf and McCoy, 2019). Therefore, a comprehensive understanding of the developmental characteristics and influencing factors of EF in preschool children is essential for fostering their healthy growth and lifelong development.

The development of EF in preschool children is influenced by multiple factors, with a healthy lifestyle being a fundamental prerequisite for cognitive growth. Among its key components, adequate physical activity (PA) and high-quality sleep play important roles in EF development. Research indicates that both acute and chronic PA can enhance cognitive flexibility and inhibitory control in preschool children, highlighting a strong association between PA and EF (Song et al., 2023). Mechanistically, PA may positively influence EF by increasing cerebral blood flow and promoting brain structural development (Hillman et al., 2014). At the same time, sleep plays a critical role in executive function development. Bruni et al. found that sleep problem can disrupt the rapidly developing prefrontal cortex, thereby impairing EF development (Bruni et al., 2020). Notably, there is also a relationship between PA and sleep. Moderate PA can improve sleep quality and extend sleep duration through mechanisms such as increasing cerebral blood flow and facilitating cortical recovery (Full et al., 2019; Passos et al., 2011).

Although previous studies have explored the relationships between PA, sleep, and EF, most have examined only two of these factors in isolation, with limited research investigating their interactions. Notably, existing studies on the potential mediating role of sleep in the relationship between PA and EF have primarily focused on children with attention deficit hyperactivity disorder (ADHD), university students, and older adults (Falck et al., 2018; Li et al., 2021; Liang et al., 2022). These studies consistently suggest that sleep mediates the association between PA and EF. However, research on this interplay remains limited in preschool children—a population experiencing both rapid EF development and a high prevalence of sleep problem.

Based on this, the present study focuses on preschool children to explore the relationships among PA, EF, and sleep problem. We aimed to investigate the pathways through which PA and sleep problem influence EF and to examine the mediating role of sleep problem in the relationship between PA and EF. The findings are expected to provide both theoretical insights and practical implications for optimizing EF development in preschool children.

Based on existing literature (Liang et al., 2022), this study hypothesized that: (1) time spent in LPA, MVPA, and TPA would be negatively associated with executive function impairments; (2) LPA, MVPA, and TPA would be negatively associated with sleep problems; (3) sleep problems would be positively associated with executive function impairments; and (4) sleep problems would partially mediate the relationship between physical activity and executive function impairments.

## 2 Method

### 2.1 Participants

**Figure 1:**
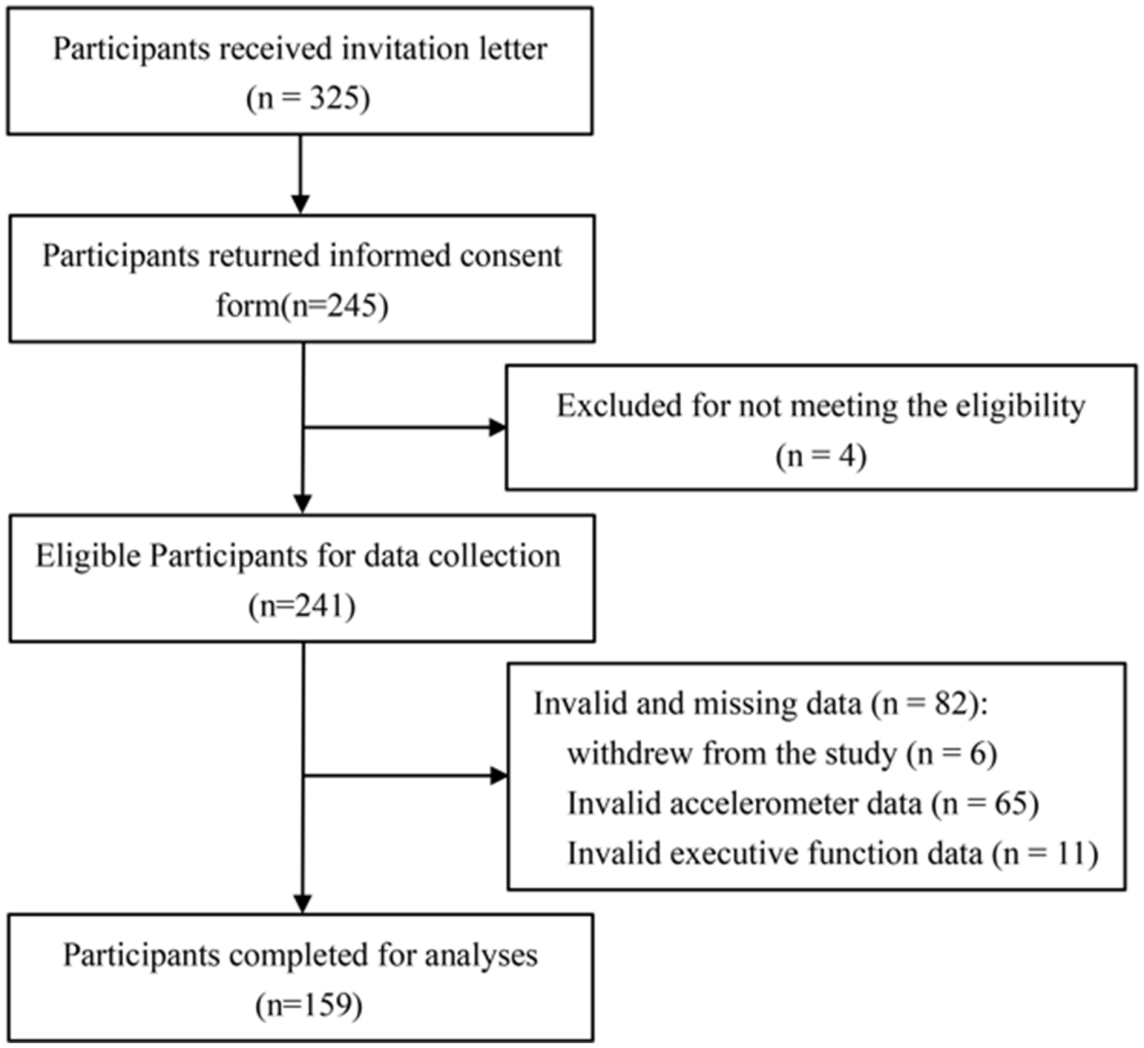
Participant inclusion flowchart.

We recruited participants through convenience sampling from four Hangzhou kindergartens between June and November 2024. A total of 325 preschool children were initially approached. Those meeting the inclusion criteria were invited to participate, with verbal assent obtained from each child and written informed consent provided by their legal guardians. Participants were allowed to withdraw from the study at any time without penalty, especially in the event of any discomfort. We applied the following exclusion criteria: (1) Invalid physical activity data due to improper accelerometer use, (2) Incomplete or missing questionnaire responses, (3) Recent restrictions on physical activity due to special circumstances, and (4) Presence of disease, including organic disorders or any mental illness. Following data screening, a final sample of 159 children (80 boys, 79 girls) was retained for analysis (see Figure 1). Sample size estimation was conducted using G*Power 3.1.9.6 software (Ceyhan and Aydin, 2024), referencing a medium effect size (Cohen, 2013), which yielded a minimum requirement of 107 participants. The final sample exceeded this threshold, ensuring adequate statistical power. This study was approved by the Ethics Committee of Hangzhou Normal University (Approval No. 2024–0801).

### 2.2 Measurement of physical activity

Physical activity levels were objectively measured using ActiGraph GT3X-BT accelerometer (Pensacola, FL, USA). The device recorded time spent in light physical activity (LPA), moderate physical activity (MPA), and vigorous physical activity (VPA), based on which moderate-to-vigorous physical activity (MVPA) and total physical activity (TPA) were calculated. Given the young age of the participants, teachers and parents assisted with wearing and removing the accelerometers. During school days, teachers checked and adjusted the devices twice daily (morning and evening). Prior to data collection, teachers and parents received detailed instructions on proper accelerometer use, including a user manual. The accelerometer was worn on the right iliac crest and remained in place at all times except during activities such as bathing and swimming. Participants wore the device continuously for 7 days (five weekdays and two weekend days). On the eighth day, researchers collected the devices and used Actilife software (version 6.13.4) to initialize, download, and analyze the data. Non-wear time was identified as periods of consecutive zero counts lasting 60 min or longer and excluded from the analysis to ensure data accuracy. Data were processed in 15-s epochs to capture the sporadic nature of preschool children’s activity patterns. To be considered valid, data had to meet the criteria of at least 480 min of wear time per day and a minimum of three valid days per week (including at least two weekdays and one weekend day). To ensure accuracy and comparability with similar studies, this study applied Butte’s intensity cut-off thresholds for preschool children, which were specifically validated for preschool-aged children (Butte et al., 2014). These thresholds define SB as 0–819 counts/min, LPA as 820–3,907 counts/min, MPA as 3,908–6,111 counts/min, and VPA as greater than 6,112 counts/min.

### 2.3 Children’s sleep health questionnaire

Sleep problems were assessed using Chinese adaptation of the Children’s Sleep Health Questionnaire (CSHQ). Originally developed by Dr. Judith Owens based on pediatric physiological characteristics (Owens et al., 2000), the CSHQ was later translated and validated in Chinese by Li et al. (2007), demonstrating its suitability for evaluating sleep patterns in healthy Chinese children. The CSHQ requires parents to recall their child’s sleep patterns over the past month and to select a typical week for reporting. The questionnaire consists of 33 scored items, each rated on a three-point Likert scale: “Usually” (5–7 times per week) = 3 points, “Sometimes” (2–4 times per week) = 2 points, “Rarely” (0–1 times per week) = 1 point. The questionnaire evaluates eight sleep domains: Sleep resistance, Sleep onset delay, Sleep duration, Sleep anxiety, Night waking, Parasomnias, Sleep-disordered breathing, Daytime sleepiness. In this study, only the total CSHQ score was used in the analysis to reflect overall sleep problems. The internal consistency reliability of the questionnaire was assessed using Cronbach’s *α* coefficient, yielding a value of 0.753, indicating good reliability. Structural validity was assessed using the Kaiser-Meyer-Olkin (KMO) measure, which produced a coefficient of 0.658, and Bartlett’s test of sphericity, which was statistically significant (*p* < 0.001), confirming the questionnaire’s robust structural validity.

### 2.4 Behavior rating inventory of executive function-preschool version

Executive function was assessed using the Behavior Rating Inventory of Executive Function-Preschool Version (BRIEF-P), originally developed by Gioia et al. (2000). This internationally validated instrument was culturally adapted for Chinese populations through Professor Wang Yufeng’s pioneering work, with subsequent psychometric validation by Dr. Lu confirming its reliability and validity within Chinese cultural contexts (Lu et al., 2017). The questionnaire consists of 63 items, categorized into: Five executive function components: inhibition, shift, emotional control, working-memory, and plan-organize. Each item is rated on a three-point Likert scale: “Never” = 1, “Sometimes” = 2, “Often” = 3. Higher total scores indicate more severe executive function impairments. In this study, the BRIEF-P was completed by parents during the same week as the accelerometer and sleep questionnaire assessments. Only the total BRIEF-P score was used in the analysis to represent overall executive function problems. The internal consistency of the BRIEF-P was assessed using Cronbach’s *α* coefficient, yielding a value of 0.954, indicating excellent reliability. Structural validity was evaluated using the Kaiser-Meyer-Olkin (KMO) measure, which produced a coefficient of 0.863, and Bartlett’s test of sphericity, which was statistically significant (*p* < 0.001), confirming the strong structural validity of the questionnaire.

### 2.5 Covariate collection

Based on relevant studies, we identified and collected covariates for model adjustment (Quan et al., 2017). Data were obtained through questionnaire surveys and physical measurements. Questionnaire-based data: Child characteristics: age, gender, only-child status, Parental characteristics: educational level, economic status. Anthropometric measurements: Height (cm) and weight (kg) were measured using a stadiometer and digital scale, respectively. Body Mass Index (BMI) was calculated as weight (in kilograms) divided by the square of height (m^2^).

### 2.6 Statistical analysis

Statistical analyses were performed using SPSS 29.0 and Mplus 8.0. Data are presented as mean ± standard deviation (M ± SD). To test for common method bias in the survey data, the control unmeasured single method latent factor approach was applied. Gender differences in physical activity, sleep problems, and executive function were analyzed using either an unpaired t-test or Mann–Whitney *U* test, depending on the data distribution. Spearman correlation analysis was used to examine the bivariate relationships between preschool children’s physical activity levels, sleep, and executive function. Structural equation modeling was conducted using Mplus 8.0 to assess the mediating role of sleep in the relationship between physical activity and executive function. The analysis followed a two-step approach: 1. Direct Effects Model: The relationship between physical activity and executive function was evaluated. 2. Mediation Model: Sleep problems were inserted as a mediating variable to examine the mediation effect. The analysis used Mean-centered Maximum Likelihood (MCML) estimation. Model fit was evaluated using the following criteria: CFI and TLI > 0.90, Root RMSEA and SRMR < 0.08. If the model fit was inadequate, Modification Indices (MI) reviewed to determine whether additional paths should be added to improve model fit.

## 3 Results

### 3.1 Common method bias

This study employed the control unmeasured single method latent factor approach to assess the risk of common method bias (Xiong et al., 2012). First, the confirmatory factor analysis (CFA) model demonstrated acceptable fit indices (*χ*^2^/*df* = 2.418, CFI = 0.874, TLI = 0.846, RMSEA = 0.094, SRMR = 0.066). Subsequently, a method latent factor was added to the original model, and the results showed minimal changes in model fit indices (△*χ*^2^/*df* = 0.111, △CFI = 0.007, △TLI = −0.012, △RMSEA = 0.002, △SRMR = 0.036), with SRMR even worsening. Therefore, the inclusion of the method latent factor did not significantly improve the model, indicating that common method bias was not a major concern in this study (Siman et al., 2015).

### 3.2 Descriptive statistics and analysis

Regarding physical activity, no significant differences between boys and girls time spent in LPA, MVPA, or TPA (*p* > 0.05; see Table 1). In terms of sleep problems, boys had significantly lower sleep duration scores compared to girls (*p* < 0.01, *Cohen’s d* = 0.43), while no significant gender differences were observed for other sleep indicators (*p* > 0.05; see Table 1). For executive function, no significant differences between boys and girls in inhibition, shift, emotional control, working-memory, Plan-organize, or total executive function scores (*p* > 0.05; see Table 1).

**Table 1:** Basic characteristics of participants.

Variable	Sex			Total (*n* = 159)
	Boys (*n* = 80), M ± SD	Girls (*n* = 79), M ± SD	*Cohen’s d*	
Physical activity				
LPA, min	197.44 ± 21.40	197.28 ± 19.47	−0.01	197.36 ± 20.53
MVPA, min	55.92 ± 11.38	58.83 ± 10.38	−0.25	57.36 ± 10.96
TPA, min	253.36 ± 29.33	256.11 ± 26.23	−0.10	254.72 ± 27.78
Sleep problems				
Bedtime resistance	9.42 ± 1.92	9.47 ± 2.08	−0.03	9.45 ± 2.00
Sleep onset delay	1.74 ± 0.74	1.78 ± 0.69	−0.06	1.76 ± 0.72
Sleep duration	4.83 ± 1.66	5.49 ± 1.40^**^	−0.43	5.16 ± 1.57
Sleep anxiety	6.14 ± 1.61	5.82 ± 1.59	0.21	5.98 ± 1.60
Night waking	3.31 ± 0.79	3.52 ± 1.12	−0.20	3.42 ± 0.97
Parasomnias	7.19 ± 1.42	7.80 ± 2.15	−0.33	7.49 ± 1.84
Sleep disordered breathing	3.31 ± 1.00	3.20 ± 0.63	0.13	3.26 ± 0.84
Daytime sleepiness	11.77 ± 2.70	12.52 ± 3.02	−0.26	12.14 ± 2.88
Total sleep problems	47.71 ± 6.03	49.61 ± 7.41	−0.27	48.65 ± 6.80
Executive function				
Inhibition	23.61 ± 5.70	23.82 ± 5.57	−0.04	23.72 ± 5.62
Emotional control	14.74 ± 3.06	15.53 ± 3.33	−0.25	15.13 ± 3.21
Shift	13.91 ± 3.95	14.24 ± 3.33	−0.08	14.08 ± 3.65
Working-memory	25.03 ± 5.36	25.86 ± 5.37	−0.16	25.44 ± 5.36
Plan-organize	14.06 ± 2.61	14.97 ± 3.43	−0.35	14.52 ± 3.07
Total executive function	91.35 ± 17.35	94.42 ± 18.34	−0.17	92.88 ± 17.86

MVPA, moderate-to-vigorous physical activity; LPA, light physical activity; TPA, total physical activity; M, mean; SD, standard deviation; ^**^means that p value is less than 0.01, comparison between boys and girls. Higher scores on the executive function and sleep problem measures indicate greater impairment. Therefore, lower scores reflect better executive function and fewer sleep-related problems.

### 3.3 Correlation analysis

From Table 2, LPA (*r* = −0.53, *p* < 0.01), MVPA (*r* = −0.61, *p* < 0.01), and TPA (*r* = −0.64, *p* < 0.01) are significantly negatively correlated with executive function scores, indicating substantial associations between physical activity levels and executive functioning in preschool children. Meanwhile, LPA (*r* = −0.27, *p* < 0.01), MVPA (*r* = −0.29, *p* < 0.01), and TPA (*r* = −0.31, *p* < 0.01) are significantly negatively correlated with sleep problems, suggesting that increased physical activity is related to better sleep health. In addition, sleep problems demonstrate a significantly positively correlation with executive function scores (*r* = 0.47, *p* < 0.01), suggesting that poorer sleep is associated with more executive function impairment.

**Table 2:** Correlation coefficients of study variables.

1		2	3	4	5	6	7	8	9	10	11	12	13
1. Age	1												
2. Gender	0.02	1											
3. Heigh	0.45^**^	0.10	1										
4. Weigh	0.41^**^	0.01	0.66^**^	1									
5. BMI	0.16^*^	−0.08	0.06	0.73^**^	1								
6. Only Child	−0.10	0.04	0.05	0.05	0.05	1							
7. Parental Education	−0.08	−0.04	0.02	−0.08	−0.07	0.05	1						
8. Parental Income	−0.13	0.02	−0.17^*^	−0.13	−0.02	0.07	0.21^**^	1					
9. LPA	0.19^*^	0.02	0.15	0.08	0.00	−0.07	0.14	−0.02	1				
10. MVPA	0.12	0.14	0.12	0.03	−0.04	0.01	0.02	−0.06	0.55^**^	1			
11. TPA	0.17^*^	0.09	0.16^*^	0.07	−0.03	−0.05	0.10	−0.06	0.93^**^	0.80^**^	1		
12. EF	−0.07	0.08	−0.06	0.01	0.06	0.04	−0.07	0.05	−0.53^**^	−0.61^**^	−0.64^**^	1	
13. SP	−0.15	0.13	−0.11	−0.10	−0.02	0.16^*^	0.00	0.14	−0.27^**^	−0.29^**^	−0.31^**^	0.47^**^	1

*p < 0.05, **p < 0.01, ***p < 0.001. BMI, Body Mass Index; LPA, Light Physical Activity; MVPA, Moderate-to-Vigorous Physical Activity; TPA, Total Physical Activity; EF, Total Executive Function Scores; SP, Total Sleep Problems Scores. Higher scores on the executive function and sleep problem measures indicate greater impairment. Therefore, lower scores reflect better executive function and fewer sleep-related problems. Values are Pearson correlation coefficients. Variables in the rows and columns are identical, as both represent the same set of variables in the correlation analysis.

### 3.4 The mediating role of sleep problems in the relationship between physical activity and executive function in preschool children

**Figure 2:**
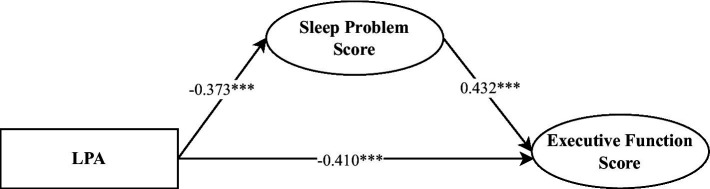
Mediation model of LPA predicting executive function.

Regarding the direct effects, LPA exhibited a significantly negatively impact on executive function scores (*β* = −0.41, *p* < 0.001), while sleep problems had a significant positive effect on executive function scores (*β* = 0.43, *p* < 0.001). In terms of indirect effects, through which LPA influences executive function scores via sleep problems was significant (*β* = −0.16, *p* = 0.001, 95% CI = [−0.26, −0.06]), with the mediation effect accounting for 28.20% of the total effect (see Table 3 and Figure 2). These findings indicate that sleep problems play a partial mediating role in the relationship between LPA and executive function in preschool children.

**Table 3:** Direct and indirect effects of LPA on executive function scores and model fit indices.

Effect/Model fit	*β*	*p*
Direct effects		
LPA → Executive function score	−0.41***	<0.001
Sleep problems Score → Executive Function Score	0.43***	<0.001
Indirect effects		
LPA → Sleep problems Score → Executive Function Score	−0.16**	0.001
Model fit indices		
CFI	0.940	
TLI	0.926	
RMSEA	0.059	
SRMR	0.062	

Higher executive function scores indicate poorer executive function performance. LPA, light physical activity. **p < 0.01, ***p < 0.001.

**Figure 3:**
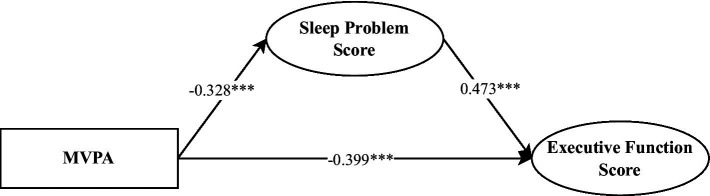
Mediation model of MVPA predicting executive function.

From the results of the direct effects, MVPA exhibited a significantly negatively impact on executive function scores (*β* = −0.40, *p* < 0.001), while sleep problems had a significantly positive effect on executive function scores (*β* = 0.47, *p* < 0.001). Regarding the indirect effects, the mediating pathway through which MVPA influenced executive function scores via sleep problems was significant (*β* = −0.16, *p* = 0.001, 95% CI = [−0.25, −0.06]), with the mediation effect accounting for 27.98% of the total effect (see Table 4 and Figure 3). These findings suggest that sleep problems partially mediate the relationship between MVPA and executive function in preschool children.

**Table 4:** Direct and indirect effects of MVPA on executive function scores and model fit indices.

Effect/Model fit	*β*	*p*
Direct effects		
MVPA → Executive function score	−0.40***	<0.001
Sleep problems Score → Executive function score	0.47***	<0.001
Indirect effects		
MVPA → Sleep problems Score → Executive function score	−0.16**	0.001
Model fit indices		
CFI	0.929	
TLI	0.911	
RMSEA	0.066	
SRMR	0.064	

Higher executive function scores indicate poorer executive function performance. MVPA, Moderate-to-Vigorous Physical Activity. **p < 0.01, ***p < 0.001.

**Figure 4:**
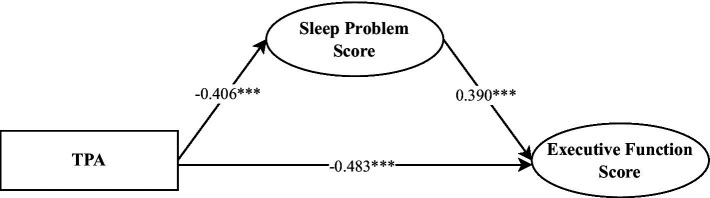
Mediation model of TPA predicting executive function.

From the results of the direct effects, TPA exhibited a significantly negative impact on executive function scores (*β* = −0.48, *p* = 0.001), while sleep problems significantly positively predicted executive function scores (*β* = 0.39, *p* < 0.001). Regarding the indirect effects, the mediating pathway through which TPA influenced executive function scores via sleep problems was significant (*β* = −0.16, *p* = 0.001, 95% CI = [−0.25, −0.06]), with the mediation effect accounting for 24.65% of the total effect (see Table 5 and Figure 4). These findings indicate that sleep problems partially mediate the relationship between TPA and executive function scores in preschool children.

**Table 5:** Direct and indirect effects of TPA on executive function scores and model fit indices.

Effect/Model fit	*β*	*p*
Direct effects		
TPA → Executive function score	−0.48***	<0.001
Sleep problems Score → Executive function score	0.39***	<0.001
Indirect effects		
TPA → Sleep problems Score → Executive function score	−0.16**	0.001
Model fit indices		
CFI	0.939	
TLI	0.925	
RMSEA	0.060	
SRMR	0.062	

**p < 0.01, ***p < 0.001.

## 4 Discussion

Given that the preschool years represent a critical period for rapid executive function development and are also marked by a high prevalence of sleep problems, this study investigated the predictive role of physical activity in executive function among preschool children and examined the mediating effect of sleep problems in this relationship. The findings indicate that LPA, MVPA, and TPA contribute to the development of executive function in preschool children, with sleep problems partially mediating the effects of these physical activity types on executive function.

### 4.1 The interrelationship among physical activity, sleep problems, and executive function in preschool children

This study demonstrated that LPA, MVPA, and TPA contribute to the development of executive function in preschool children, which aligns with the findings of Bezerra et al. (2021) and Lau et al. (2024). Previous research has shown that physical activity, regardless of intensity (low-intensity, moderate-to-vigorous intensity, or total physical activity), positively influences executive function and its core components, including working memory, inhibitory control, and cognitive flexibility. From a mechanistic perspective, physical activity may enhance executive function by stimulating the release of neurotrophic factors, promoting angiogenesis and neurogenesis, modifying brain structure and functional connectivity, and optimizing brain activation patterns (Chen and Nakagawa, 2023; Latomme et al., 2022). During fundamental physical activities such as running, jumping, and throwing, children must rapidly process and store information while issuing motor commands to adjust their movements in real time. Throughout this process, they continuously regulate posture, movement speed, rhythm, and trajectory while suppressing irrelevant information to optimize goal-directed behavior. This complex sensorimotor regulation process enhances working memory, inhibitory control, and cognitive flexibility. However, Willoughby et al. reported a negative association between MVPA and executive function and no significant correlation between LPA and executive function (Willoughby et al., 2018), contrasting with the present study’s findings. This discrepancy may be partly attributed to methodological differences in the assessment of executive function. Willoughby et al. utilized EF Touch, a computerized performance-based battery of EF tasks, while the current study employed the BRIEF-P, a parent-reported questionnaire assessing children’s everyday executive functioning in naturalistic settings. While both methods are valid, they capture different facets of EF: performance tasks assess optimal functioning under structured conditions, whereas questionnaires reflect typical behaviors across contexts. These differences in measurement approaches may contribute to inconsistent findings across studies.

Additionally, this study found that higher levels of LPA, MVPA, and TPA were significantly associated with fewer sleep problems in preschool children, consistent with prior research (Hu et al., 2024). Physical activity may mitigate sleep problems by regulating core body temperature, lowering resting heart rate, enhancing neurotrophic factor secretion, strengthening cortical inhibition, improving emotional states, and modifying hippocampal volume (De Nys et al., 2022; Tseng et al., 2020). Furthermore, this study found that sleep problems were significantly associated with poorer executive function in preschool children, in line with previous research (Turnbull et al., 2013). Studies have shown that children with executive function impairments are more likely to experience sleep disturbances compared to their typically developing peers (Turnbull et al., 2013). Research by Shay et al. further indicated that chronic sleep deprivation can lead to cerebral hypoxia (Shay et al., 2014), which in turn compromises overall brain function and synaptic plasticity, slows information processing speed, and manifests as reduced attentional stability, dizziness, memory deficits, and impaired focus. These findings underscore the importance of early identification and management of sleep problems in preschool children, as improving sleep quality may serve as a feasible intervention target to support cognitive development during this critical developmental stage.

### 4.2 The mediating role of sleep problems in the relationship between physical activity and executive function

This study further examined the relationships between LPA, MVPA, TPA, and executive function, and found that sleep problems partially mediated these associations. These findings are consistent with prior research conducted on adults and children with ADHD (Falck et al., 2018; Li et al., 2021; Liang et al., 2022). Existing evidence suggests that physical activity enhances executive control and memory consolidation by increasing slow-wave sleep. Reduced nocturnal awakenings and improved sleep continuity contribute to a higher proportion of slow-wave sleep, which is predominantly characterized by neural synchronization in the prefrontal cortex. This synchronization may strengthen synaptic functions within networks essential for executive function (Wilckens et al., 2016). Such a mechanism may explain how physical activity supports early brain maturation and executive function development in preschool children by improving sleep quality. Another potential explanation for the mediating role of sleep problems involves cerebral blood flow regulation. Physical activity optimizes cerebral blood flow dynamics (Querido and Sheel, 2007), enhancing brain oxygenation during the sleep–wake cycle, which in turn contributes to improvements in executive function. Notably, the indirect effects of sleep problems accounted for 28.20, 27.98, and 24.65% of the total effects of LPA, MVPA, and TPA on executive function, respectively. The comparable mediation proportions indicate that even LPA may contribute to improving executive function by enhancing sleep quality. This suggests that LPA could be considered as a feasible and accessible component in interventions aimed at supporting cognitive development in preschool children. While sleep problems served as a mediating factor, the well-documented benefits of physical activity on executive function are mediated through multiple biological and cognitive pathways, involving mechanisms at the molecular, cellular, and systemic levels. Sleep problems represent just one of the behavioral factors mediating this relationship and cannot fully account for the effects of physical activity on executive function. Other potential mediators include brain-derived neurotrophic factor (Quan et al., 2017) and physical fitness index (Wu et al., 2025), both of which have been associated with cognitive functioning in children. Therefore, further research is needed to explore the broader and more complex mechanisms through which physical activity enhances cognitive function, particularly during the preschool years.

## 5 Educational recommendations and limitations

### 5.1 Implementing diverse strategies to increase physical activity and enhance executive function in preschool children

Executive function is crucial for preschool children’s development and is highly malleable. Early training during the preschool years can yield significant benefits. However, in China, many preschool teachers and parents have limited awareness of the concept of executive function, highlighting the need to raise their awareness to improve educational practices and strategies (Fei et al., 2019). This study found that regular physical activity was found to be significantly associated with better executive function and fewer sleep problems in preschool children, suggesting its potential importance for early cognitive and sleep health. However, due to safety concerns, preschool teachers often hesitate to organize or limit the frequency of MVPA (Chang et al., 2020; Copeland et al., 2012).

To effectively support executive function development in preschool children, both teachers and parents should adopt a multifaceted approach to increase children’s physical activity. (1) Optimizing Preschool Physical Education Programs. Preschools should continue to implement the “Health Domain” objectives outlined in the Chinese Guidelines for the Learning and Development of Children Aged 3–6 while addressing the practical challenges of organizing moderate-to-vigorous physical activities. Further exploration of the positive effects of MVPA on preschool children’s executive function is necessary. Programs such as Anji Play and adventure-based activities can be effective approaches (Wu et al., 2024). (2) Introducing Structured and Engaging Physical Activities. Drawing from domestic and international research, structured and engaging physical activities have been validated as effective strategies for promoting children’s physical and cognitive development (Xiong et al., 2019). Preschool children can participate in various motor skill training activities, such as soccer games, tennis games, and taekwondo training (Chang et al., 2013; Mulvey et al., 2018; Robinson et al., 2016). However, further investigation is required to determine the effects of different physical activity designs (e.g., type, intensity) on executive function development in preschoolers. (3) Encouraging Parental Support for Movement Development. Parents should recognize the importance of both cognitive and motor development in their children. Creating a positive home environment that encourages physical activity is essential. Additionally, parental support for physical activity has been shown to facilitate executive function development in children aged 3–6 years.

### 5.2 Enhancing executive function through high-quality sleep via home-school collaboration

This study found that physical activity enhances executive function in preschool children by improving sleep quality, with sleep problems acting as a partial mediator in this relationship. However, sleep issues among young children have become a global public health concern, with the preschool years marking a critical period for the onset and peak of many sleep-related difficulties (Licis, 2017). The World Health Organization’s Guidelines on Physical Activity, Sedentary Behavior, and Sleep for Children Under 5 Years of Age emphasize that achieving high-quality sleep and engaging in sufficient physical activity are essential for optimal growth and development (WHO, 2019). These guidelines provide a valuable framework for enhancing executive function through improved sleep quality.

Research suggests that environmental factors, such as household noise levels, can significantly affect children’s sleep quality and, in turn, impact early executive function development (Xing et al., 2022). Based on these findings, the following recommendations are proposed: (1) Creating a Quiet and Comfortable Sleep Environment. A calm and cozy sleep setting should be established to facilitate high-quality sleep for preschool children. (2) Ensuring Sufficient Sleep Duration. Parents should ensure that children receive adequate sleep daily, with nighttime sleep lasting at least 10 h. On weekends and holidays, maintaining a consistent sleep schedule is crucial to preserving biological rhythms and fostering healthy sleep habits. (3) Strengthening Home-School Communication on Sleep Management. Parents and preschool teachers should maintain close communication and cooperation to monitor children’s sleep conditions (Bird et al., 2023). By regularly discussing sleep quality and routines at home and in preschool, both parties can work together to provide a stable and healthy sleep environment. This collaborative approach lays a solid foundation for the sustainable development of preschool children’s executive function.

### 5.3 Limitations

While this study provides preliminary evidence of the mediating role of sleep problems in the relationship between physical activity and executive function in preschool children, several limitations should be acknowledged. First, sleep problems and executive function were assessed through parent-reported questionnaires. Although these measures have demonstrated reliability and validity, they remain subjective. Future research should incorporate objective assessments, such as actigraphy or standardized cognitive testing, to obtain more precise and comprehensive data. Second, the study employed a cross-sectional design, limiting the ability to establish causal relationships. Longitudinal studies are necessary to validate the observed associations and assess the long-term effects of physical activity on executive function via sleep improvements. Third, the sample was drawn exclusively from Hangzhou using a convenience sampling method, which may introduce selection bias and limit the generalizability of the findings. Future research should aim to collect data from diverse regions and cultural backgrounds to enhance the applicability and external validity of the results. Lastly, several potential confounding variables, such as socioeconomic status, dietary habits, parenting style, and screen exposure, were not controlled for in this study. These factors may influence both sleep quality and executive function, potentially biasing the observed mediation effects. Future studies should consider controlling for these confounders to better isolate the unique contribution of physical activity and sleep.

## 6 Conclusion

Higher levels of LPA, MVPA, and TPA were significantly associated with better executive function and fewer sleep problems in preschool children. Sleep problems play a partial mediating role in the relationship between physical activity and executive function, highlighting the importance of promoting physical activity to improve both sleep health and cognitive development. Preschool teachers and parents should implement structured physical activity interventions to optimize executive function by fostering better sleep quality. Future research should prioritize longitudinal and intervention studies to better elucidate the mechanisms and long-term effects of different types and intensities of physical activity on executive function to provide more targeted intervention strategies. Promoting early-life physical activity is not only vital for individual executive function and sleep health but also essential for cultivating a healthier, more productive society, serving as a call to action for schools, families, health professionals.

## Data Availability

The raw data supporting the conclusions of this article will be made available by the authors, without undue reservation.
